# A Rare Case of a Heterotopic Sacrococcygeal Glial Nodule

**DOI:** 10.7759/cureus.66085

**Published:** 2024-08-03

**Authors:** William Moore, Siya N Setty, Allan E Stolarski, Christopher Muratore, Nayana Somayaji

**Affiliations:** 1 Radiology, Boston University School of Medicine, Boston, USA; 2 Surgery, Boston Medical Center, Boston, USA; 3 Pediatric Surgery, Boston University School of Medicine, Boston, USA

**Keywords:** mri, ultrasound, pediatric, gfap, afp, sacrococcygeal, heterotopic glial nodule

## Abstract

Heterotopic glial nodule is a rare congenital non-neoplastic lesion that is characterized by ectopic brain tissue. It has occasionally been reported to affect areas such as the nose and face. The report presents a rare case of sacrococcygeal heterotopic glial nodule. Although teratomas are the most common neoplasms in this region, clinicians and radiologists should consider heterotopic glial nodule as a differential diagnosis, despite rarity and nonspecific imaging findings. Histopathology plays a crucial role in diagnosis, which intensely stains with glial fibrillary acidic protein and S-100.

## Introduction

Ectopic glial tissue is a benign congenital lesion found primarily in infants. It is characterized by the presence of glial tissue in an aberrant location outside the central nervous system [1]. The most commonly reported location of heterotopic tissue is in the nose, pharynx, and lungs [1]. It is very rare in the sacrococcygeal region, and to our knowledge, only three cases have been reported in the literature. The most common differential diagnosis for solid lesions is sacrococcygeal teratoma, which can be malignant in 12% of cases [2]. The intrinsic characteristics and a normal serum alpha-fetoprotein should prompt one to consider this as a differential diagnosis, despite the rarity of the heterotopic lesion.

This case report presents a unique case of a 10-month-old baby girl born with a palpable sacral nodule, which posed a diagnostic challenge on imaging. Through a comprehensive review of the literature and detailed analysis of this case, the report aims to shed light on the clinical presentation, diagnostic approach on imaging, management, and histopathological considerations associated with this entity.

## Case presentation

**Figure 1:**
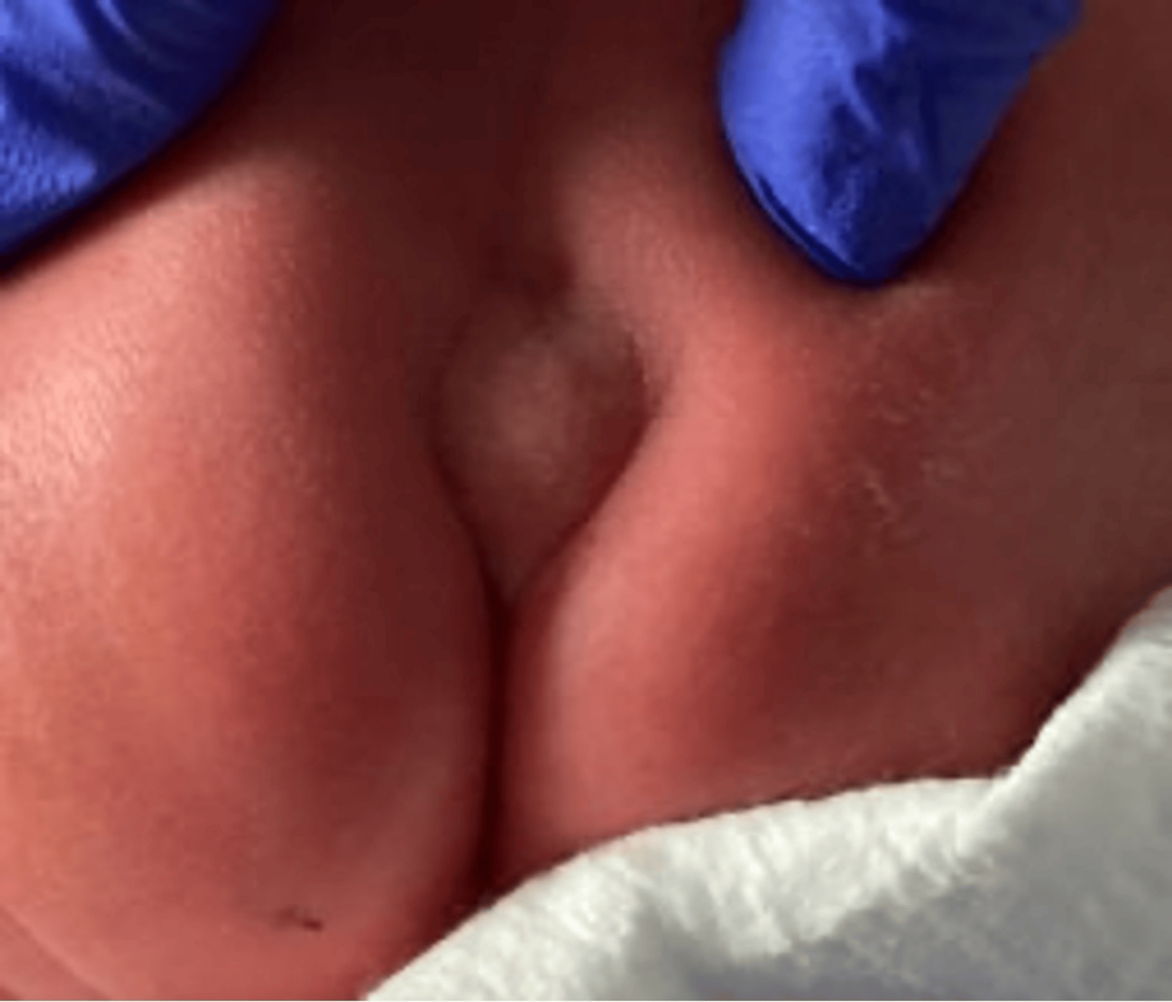
Nodule in the sacrococcygeal region

A 10-month-old baby girl presented with a palpable sacral mass and shallow sacral dimple at the gluteal cleft, which had been present since birth. The peripartum course was notable for intra-uterine growth restriction, microcephaly, and COVID-positive pregnancy. Serum alpha-fetoprotein (was slightly elevated to 26.3 ng/mL (normal range: 0-8 ng/mL). On physical exam, a nodular, firm, approximately 1 cm palpable mass at the gluteal cleft was noted (Figure 1). No induration, sinus opening, or tract was appreciated. The patient was subsequently referred for an ultrasound.

**Figure 2:**
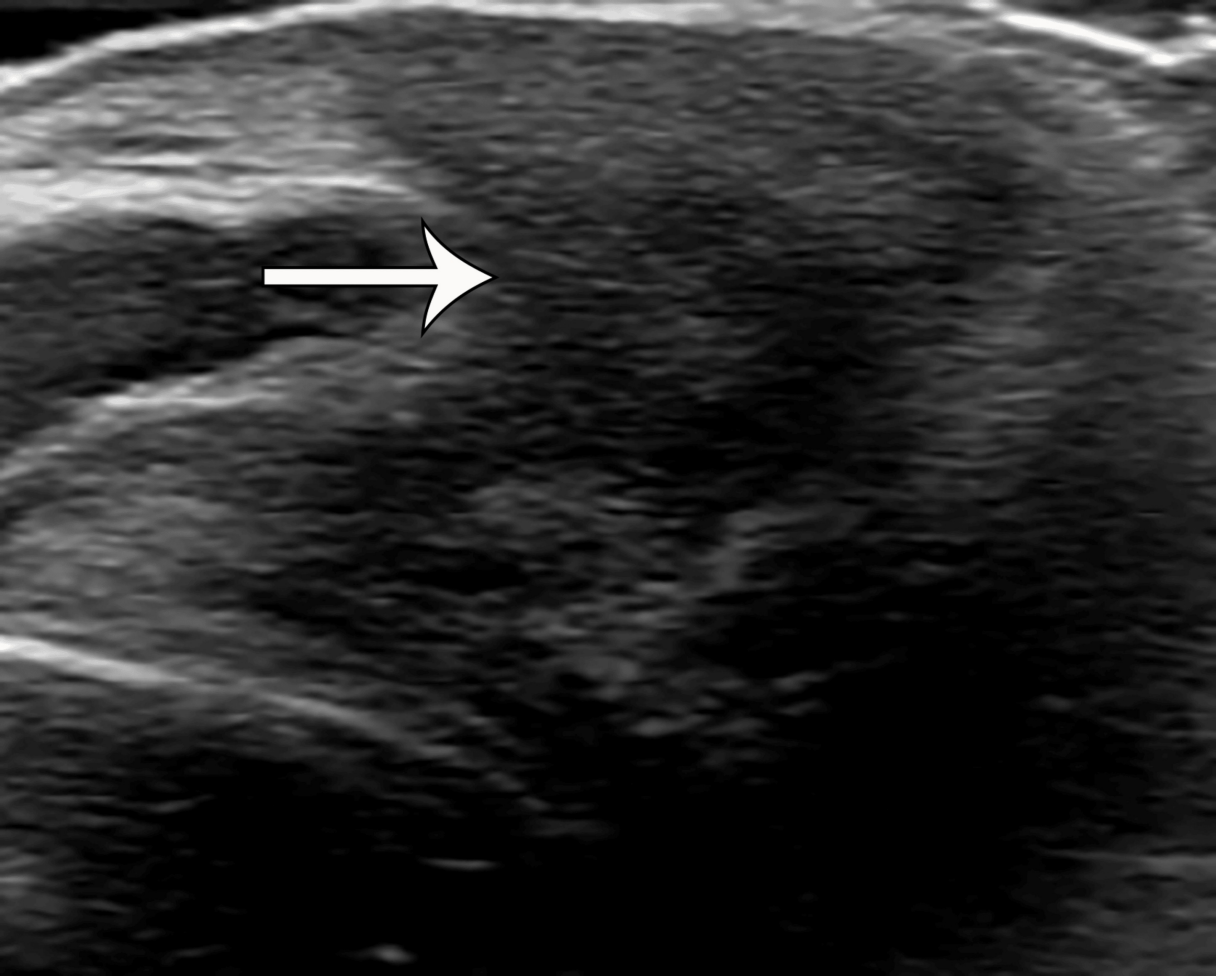
Ultrasound showing an ill-defined, hypoechoic lesion (white arrow) in the sacrococcygeal subcutaneous region with calcifications or cystic components

**Figure 3:**
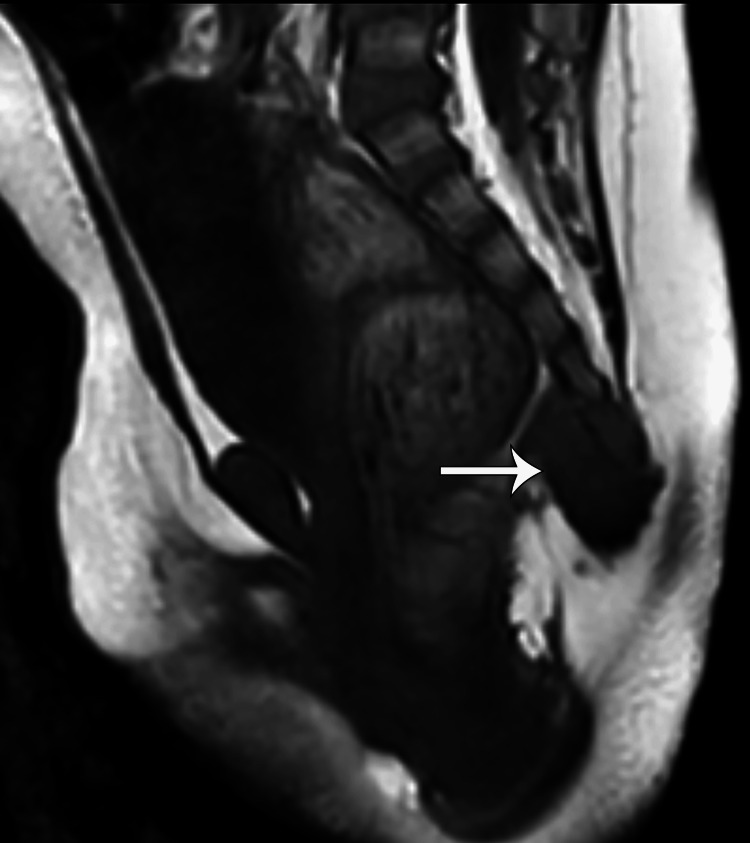
Pre-contrast T1-weighted sagittal MRI image showing a homogeneously hypointense lesion (white arrow) abutting the coccyx. No intrinsic areas of T1 hyperintense to suggest fat, calcification, or hemorrhage. No osseous anomaly or definitive erosion. Clear fat plane between lesion and rectum

**Figure 4:**
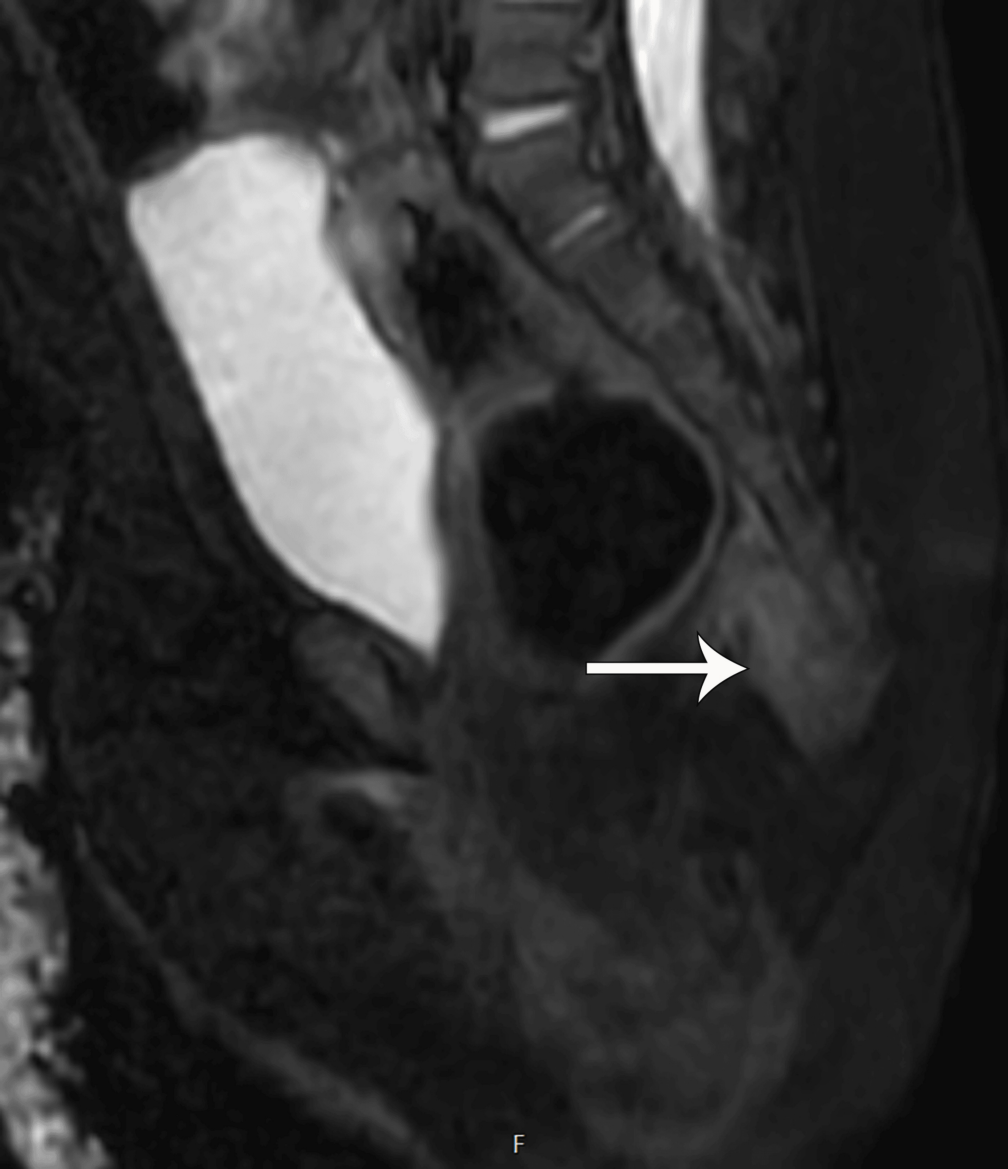
The lesion on T2-weighted STIR sagittal MRI image showing homogeneous mild hyperintensity (white arrow). No cystic components or necrosis. No bone marrow edema pattern of the coccyx

**Figure 5:**
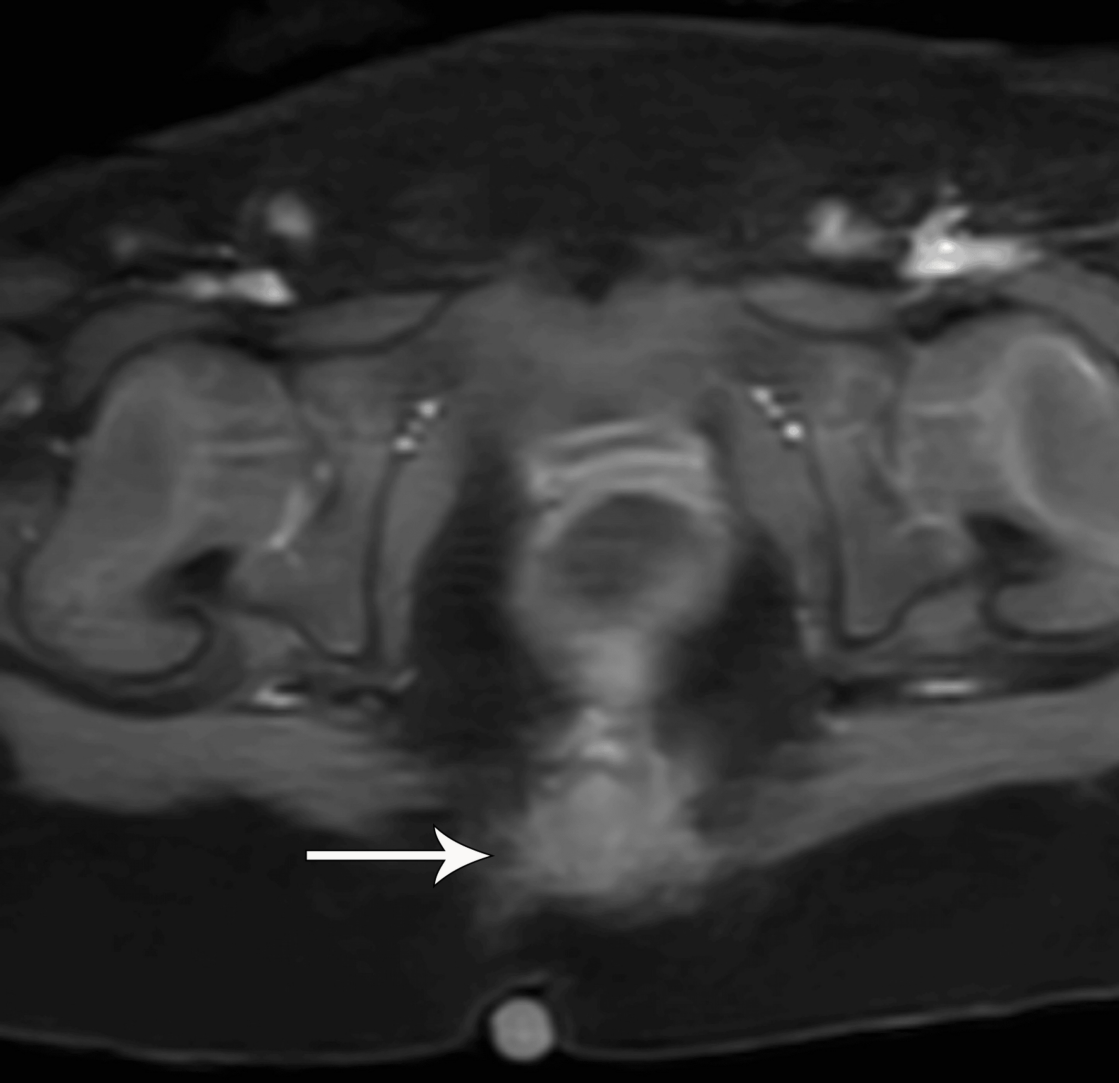
Post-contrast T1-weighted fat-saturated axial MRI image showing homogenous enhancement (white arrow)

Ultrasound of the spine performed at birth showed a hypoechoic lesion inferior to the coccyx extending from the sacrococcygeal region to the superficial skin. No calcification, cystic component, or intrinsic vascularity was seen (Figure 2). An MRI was recommended for further characterization of the lesion, which showed a homogeneous T1 hypointense, mild T2 hyperintense, and homogeneously enhancing lesion without areas of fat, necrosis, or calcifications. The lesion was also seen abutting the tip of the coccyx, which did not show an abnormal bone marrow edema pattern or any osseous anomaly or erosion. There was a clear fat plane between the lesion and the posterior wall of the rectum, and no lymphadenopathy was seen (Figures 2-5). Given the location and elevated AFP, a presumptive diagnosis of an atypical teratoma was given.

**Figure 6:**
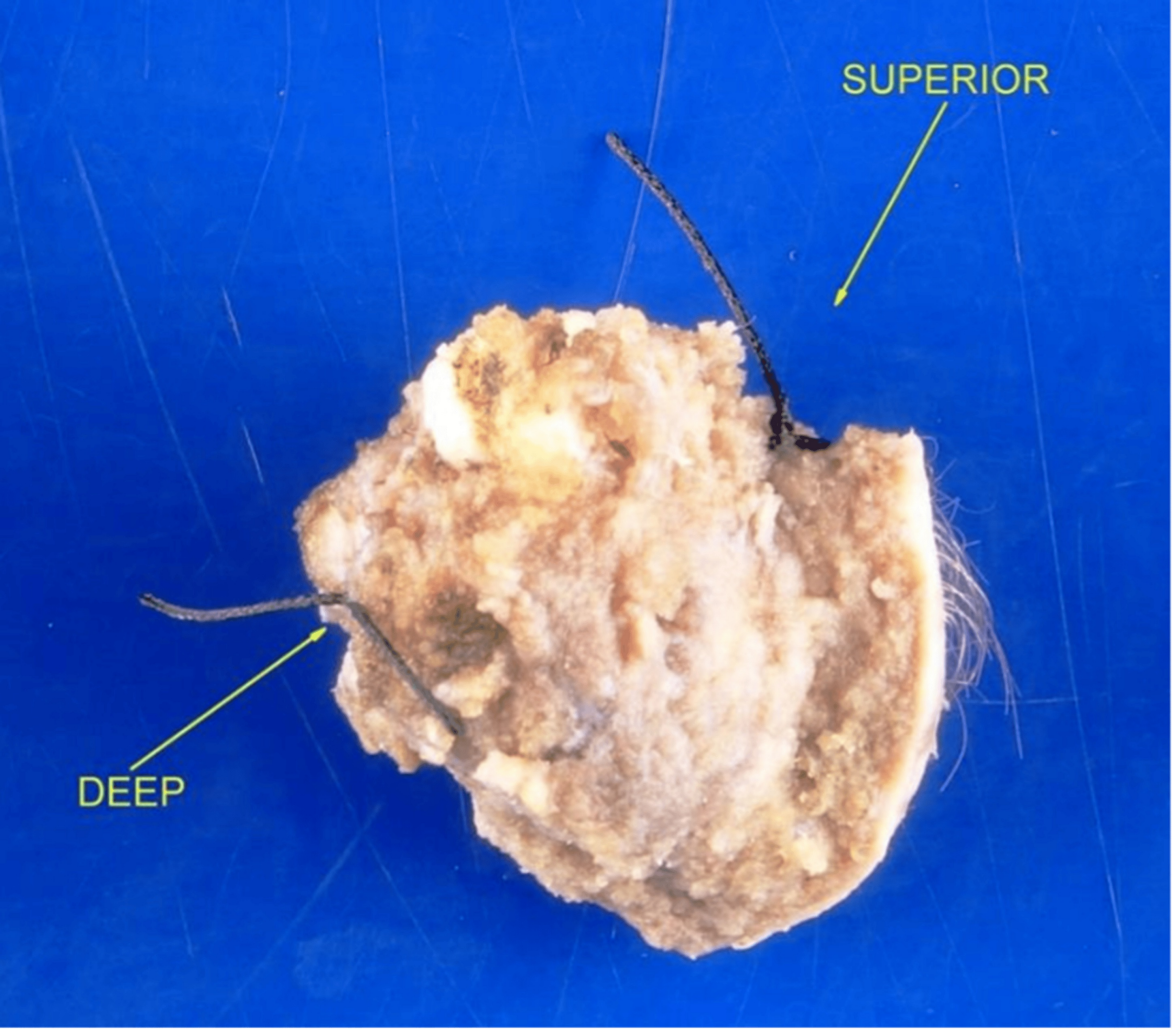
Gross specimen of the excised mass with the adjacent coccyx

**Figure 7:**
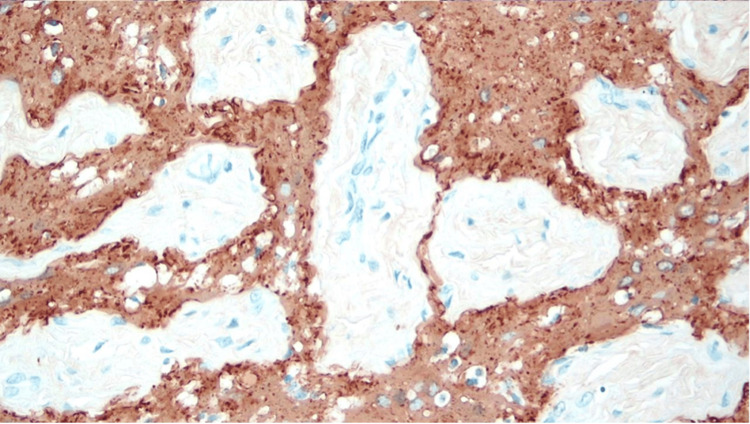
Sections demonstrating glial tissue with admixed fibrous tissue and skeletal muscle. Stained with glial fibrillary acidic protein and S-100. No features of malignancy and no fat or calcifications

MRI showed a lesion measuring 2.0 x 2.4 x 2.3 cm (AP x TV x CC). The patient was subsequently taken for total resection of the lesion (Figure 6). Final pathology reported a heterotopic glial nodule that stained with glial fibrillary acidic protein (GFAP) and S-100 but not with neurofilament protein (NFP) or epithelial membrane antigen (EMA) suggestive of glial cells with neural crest origin (Figure 7). No adjuvant chemotherapy/radiotherapy was necessary due to benignity. 

**Figure 8:**
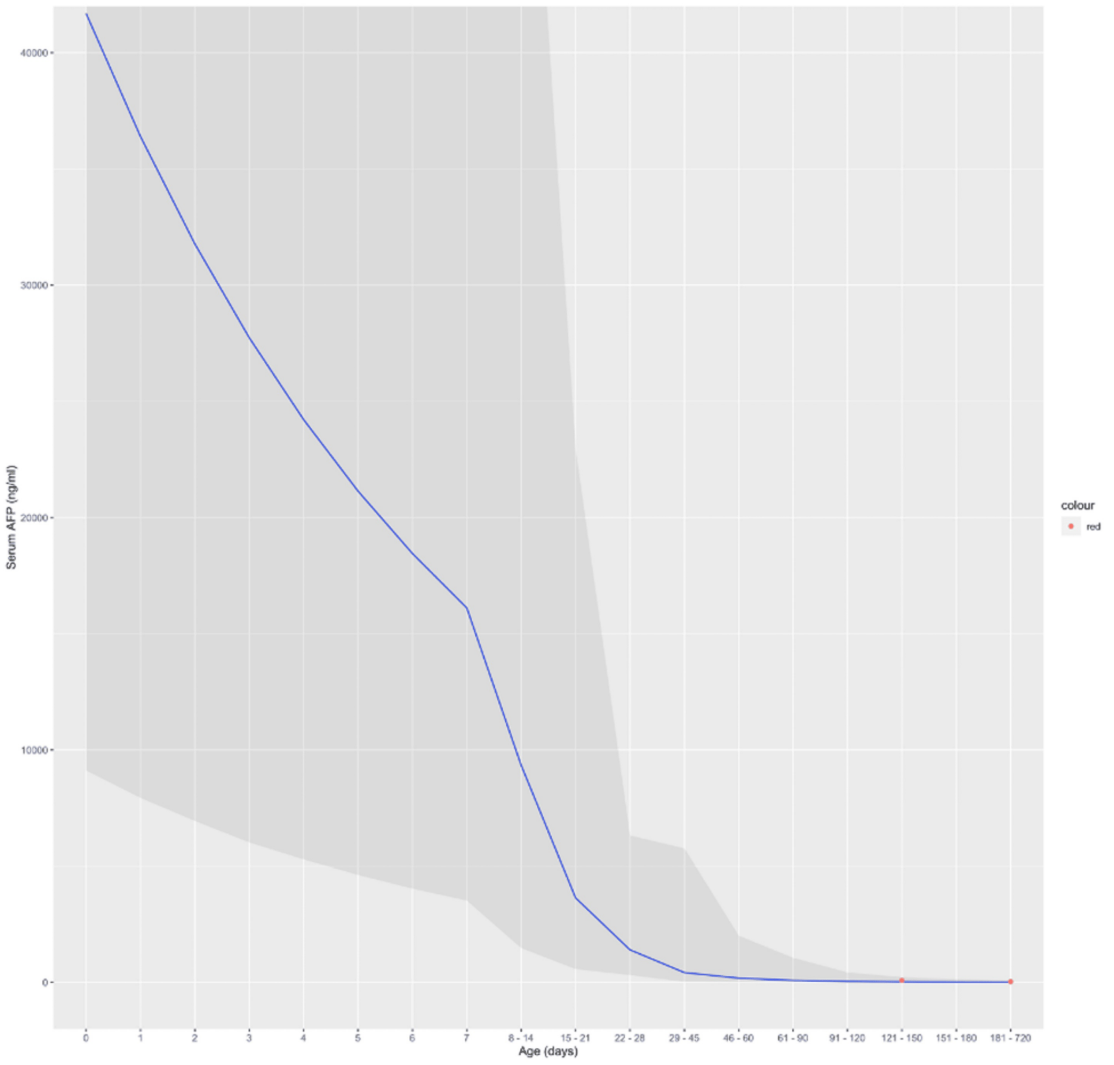
Graph demonstrating the mean serum AFP (ng/mL) as the blue line surrounded by the darker shaded 95% CI. The red dots represent the patients' measured serum alpha-fetoprotein values at 121-150 and 181-720 days of life (DOL)

Blohm et al. [3] analyzed the serum AFP levels obtained from venous or capillary blood samples from over 390 different children at various time points ranging from birth to 24 months of life. The blue line in the figure below represents the mean serum AFP (ng/mL) at various time ranges in full-term infants without factors known to be associated with elevated AFP values (premature birth, liver disease, hyperbilirubinemia). The darker shaded region surrounding the blue line is the 95% CI for the mean serum AFP levels. The red dots are the serum AFP levels that were obtained from the patient at 139 and 251 days of life (DOL), which correspond to serum AFP values of 78.2 and 26.3 ng/mL, respectively (Figure 8).

## Discussion

The presence of mature glial tissue outside of the central nervous system is a well-documented occurrence. The most common location of non-neoplastic heterotopic neural tissue is in the nasal region, sometimes referred to as a nasal glioma, although it has been noted less commonly in other sites, including the scalp, orbit, middle ear, pharynx, and lungs [1]. These typically present in the newborn period and can be associated with airway obstruction and feeding difficulties. They are more common in females and are not typically associated with other congenital defects.

Sacrococcygeal teratoma (SCT) is the foremost differential diagnosis to be considered in a newborn with a palpable/visible mass in the gluteal region. SCTs are rare but are the most common solid tumor in this region [4]. These are varied in appearance on imaging based on the contents of the lesion. The majority have mixed solid/cystic components with the presence of calcification and fat. The solid components of the lesion typically enhance, and the presence of hemorrhage and necrosis indicates malignancy.

Other neoplasms that are not pertinent to this age group are ganglioneuroma, chordoma, and subcutaneous sacrococcygeal myxopapillary ependymoma. Ganglioneuromas are rare, benign, and slow-growing tumors of the sympathetic nervous system originating from neural crest cells with an age range of 5-70 years [5]. They are predominantly heterogeneous on T2 and post-contrast imaging. Fine and speckled calcification may be observed in 20% of patients [5]. Chordomas are also rare tumors that are typically hyperintense on T2-weighted images. The most common sites for chordomas are the sacrococcygeal area in adults, whereas spheno-occipital synchondrosis is the most common location in children [6]. The lesion in the infant was only mildly T2 hyperintense, so these rare neoplasms were less likely based on age, location, and T2 characteristics. Subcutaneous sacrococcygeal myxopapillary ependymoma has a median age of 36 years and is T2 hyperintense and enhanced on contrast administration [7].

Other differential diagnoses in the age group that did not fit clinically or on imaging are subcutaneous lipoma, meningomyelocele, pilonidal cyst, and other infectious/inflammatory conditions [2].

The lesion in the patient did not exhibit typical characteristics of well-established entities in this location and in this age group.

Serum AFP levels in the patient were slightly elevated. Alpha-fetoprotein (AFP) is an embryo-specific protein that is synthesized beginning at one month of embryonic life in the yolk sac and liver, followed by synthesis predominantly in the liver. Elevated maternal serum and amniotic fluid AFP levels are common diagnostic indicators of potential fetal abnormalities, such as neural-tube and ventral wall (omphalocele, gastroschisis) defects. AFP is also a biomarker for neonatal tumors such as yolk sac and non-seminomatous germ cell tumors. AFP levels vary greatly in utero, reaching a maximum level in fetal serum of 3 mg/mL at three months of gestation after which it drops linearly approaching birth [8]. While the serum concentration decreases following the first trimester, AFP synthesis actually continues to increase until 20 weeks of gestation [8]. The decrease in serum concentration is due to the growth of the fetus and expansion of effective circulating volume effectively diluting serum AFP concentrations. After birth, AFP levels decline rapidly and typically reach normal adult levels by one to two years of life [9]. The serum AFP measurements taken at 139 DOL and 251 DOL indicated elevated values based on the laboratory values, however, comparison with the results from the Blohm study is captured within the 95% CI. Figure 8 demonstrates that the AFP values found in the patient were not significantly higher than the mean serum AFP values; thus, the patient had a healthy decrease of AFP over the infancy period, ruling out SCT and other germ cell tumors.

In histopathology, glial heterotopias are characterized by varying proportions of neurons and glia with varying degrees of fibrosis and are frequently associated with inflammation (40% of cases). Mason’s trichrome stain combined with S-100 protein and glial fibrillary acidic protein can be most helpful in accentuating the neural tissue in the background fibrosis. Neuron-specific enolase may also be used [10].

To our knowledge, only three cases of glial heterotopia have been reported so far in the sacrococcygeal region [11], and the current case is the fourth one. The first two cases were reported in 2013 and 2016 in full-term baby girls in Sri Lanka and Nepal, respectively. The third case was a male infant reported in 2019 from Afghanistan. All of these were large lesions and at least 7 cm in size in the greatest dimension, in contrast to our lesion, which was 2.4 cm in its greatest dimension. All the prior reported cases had solid and cystic components with associated flecks of calcification, which our case did not have. The 2016 and 2019 cases also had a hemorrhage on histopathology, which was not present in our case. The diagnosis of glial heterotopia depends on histopathological examination, and preoperative misdiagnosis is reported to be as high as 99% [1].

## Conclusions

Glial heterotopia is a rare occurrence but should still be considered in the differential diagnosis of a gluteal mass when the typical findings of SCT are not observed. These are isolated entities, and no association to other congenital anomalies has been reported. Reported elevated serum-AFP levels should be statistically significant in order to warrant a diagnosis of SCT.
